# Supplementary material for: Dynamic image-informed selection of biomechanical tumor growth models
Source: Biomech Model Mechanobiol. 2026 Aug 31;25(5):97. doi: 10.1007/s10237-026-02121-2 (PMC13529865; doi:10.1007/s10237-026-02121-2)
Supplement: Supplementary file 1 [file 10237_2026_2121_MOESM1_ESM.pdf]

Supplementary Information: Dynamic Image-Informed Selection of Biomechanical Tumor Growth Models

Abdullah Al Noman, Pratyush Kumar Singh, David A Hormuth, II and

Danial Faghihi

1 Laplace approximation for scalable Bayesian inference

This section summarizes the Laplace approximation of the posterior distribution for scalable Bayesian inference with high-dimensional, spatially varying parameters. For clarity, we suppress the time index and write the formulation for a single Bayesian update. In the main manuscript, this update corresponds to one assimilation step, where the posterior is conditioned on all imaging data available up to the current observation time. The Laplace approximation assumes the posterior as a Gaussian distribution centered at the maximum a posteriori (MAP) estimate and posterior covariance matrix $\mathbf{\Gamma}_{\text{post}}$, such that $\pi_{\text{post}}(\boldsymbol{\theta} \mid d) \approx \mathcal{N}(\boldsymbol{\theta}_{\text{MAP}}, \mathbf{\Gamma}_{\text{post}})$. The MAP estimate is defined as the minimizer of the negative log-posterior,

$$\boldsymbol{\theta}_{\text{MAP}} := \arg\min_{\boldsymbol{\theta}} \left\{ \frac{1}{2\sigma^2_{\text{noise}}} \int_{\Omega_0} \left[\phi(\mathbf{X}) - d(\mathbf{X})\right]^2 d\mathbf{X} + \frac{1}{2}\left\|\boldsymbol{\theta} - \bar{\boldsymbol{\theta}}\right\|^2_{\mathbf{\Gamma}^{-1}_{\text{pr}}} \right\}, \tag{S.1}$$

where ϕ denotes the model predicted tumor volume fraction and $\bar{\boldsymbol{\theta}}$ and $\mathbf{\Gamma}_{\text{pr}}$ are the mean and covariance of the Gaussian random field prior. Here, d may denote either a single MRI or the cumulative data vector $d^{(1:k)}$ used at one assimilation step in the main manuscript. Under the Laplace approximation, the posterior covariance is given by the inverse Hessian of the negative log-posterior evaluated at the MAP point,

$$\mathbf{\Gamma}_{\text{post}} = \boldsymbol{H}^{-1}(\boldsymbol{\theta}_{\text{MAP}}) = \left(\boldsymbol{H}_{\text{misfit}}(\boldsymbol{\theta}_{\text{MAP}}) + \mathbf{\Gamma}^{-1}_{\text{pr}}\right)^{-1}, \tag{S.2}$$

where $\boldsymbol{H}(\boldsymbol{\theta}) := -\nabla^2_{\boldsymbol{\theta}\boldsymbol{\theta}} \log \pi_{\text{post}}(\boldsymbol{\theta} \mid d)$ and $\boldsymbol{H}_{\text{misfit}}(\boldsymbol{\theta}) := -\nabla^2_{\boldsymbol{\theta}\boldsymbol{\theta}} \log \pi_{\text{like}}(d \mid \boldsymbol{\theta})$, denotes data-misfit Hessian. In practice, the posterior covariance is constructed using the Gauss–Newton approximation of the data-misfit Hessian, which is symmetric positive semi-definite and is standard in PDE-constrained Bayesian inverse problems.

1.1 Low-rank approximation of the posterior covariance

For spatially discretized parameter fields, the parameter dimension N_p is typically very large equivalent for number of nodes in the finite element mesh for each biomechanical parameter, making explicit construction and inversion of the Hessian matrix in (S.2) computationally intractable, i.e., *curse of dimensionality.* To enable efficient inference, we exploit the low effective rank of the data-misfit Hessian induced by limited and noisy observations [1–3]. We introduce the prior-preconditioned data-misfit Hessian,

$$\widehat{\boldsymbol{H}}_{\text{misfit}} := \boldsymbol{\Gamma}_{\text{pr}}^{1/2} \boldsymbol{H}_{\text{misfit}} \boldsymbol{\Gamma}_{\text{pr}}^{1/2}, \tag{S.3}$$

which is symmetric positive semi-definite. Using (S.2), the posterior covariance can be written as,

$$\boldsymbol{\Gamma}_{\text{post}} = \boldsymbol{\Gamma}_{\text{pr}}^{1/2} \left(\widehat{\boldsymbol{H}}_{\text{misfit}} + \boldsymbol{I} \right)^{-1} \boldsymbol{\Gamma}_{\text{pr}}^{1/2}.$$

The dominant data-informed subspace is obtained from the generalized eigenvalue problem

$$\boldsymbol{H}_{\text{misfit}} \boldsymbol{W} = \boldsymbol{\Gamma}_{\text{pr}}^{-1} \boldsymbol{W} \boldsymbol{\Lambda},$$

where $\boldsymbol{\Lambda} = \text{diag}(\lambda_i)$ contains the generalized eigenvalues and the columns of $\boldsymbol{W}$ are $\boldsymbol{\Gamma}_{\text{pr}}^{-1}$-orthonormal eigenvectors, i.e., $\boldsymbol{W}^T \boldsymbol{\Gamma}_{\text{pr}}^{-1} \boldsymbol{W} = \boldsymbol{I}$. Defining $\boldsymbol{V} := \boldsymbol{\Gamma}_{\text{pr}}^{-1/2} \boldsymbol{W}$, it follows that the columns of $\boldsymbol{V}$ are orthonormal eigenvectors of the prior-preconditioned Hessian, $\widehat{\boldsymbol{H}}_{\text{misfit}} \boldsymbol{V} = \boldsymbol{V} \boldsymbol{\Lambda}$. In many PDE-constrained Bayesian inverse problems, the generalized eigenvalues $\{\lambda_i\}$ decay rapidly, so that only the leading $r \ll N_p$ modes are required to accurately represent the data-informed subspace. Let $\boldsymbol{V}_r$ and $\boldsymbol{\Lambda}_r$ denote the truncated eigenvectors and eigenvalues, respectively. Then,

$$\left(\widehat{\boldsymbol{H}}_{\text{misfit}} + \boldsymbol{I} \right)^{-1} \approx \boldsymbol{I} - \boldsymbol{V}_r \boldsymbol{D}_r \boldsymbol{V}_r^T, \qquad \boldsymbol{D}_r = \text{diag}\left(\frac{\lambda_i}{1 + \lambda_i} \right). \tag{S.4}$$

Substituting this approximation into the Laplace covariance expression yields [2, 3]

$$\boldsymbol{\Gamma}_{\text{post}} \approx \boldsymbol{\Gamma}_{\text{pr}} - \boldsymbol{W}_r \boldsymbol{D}_r \boldsymbol{W}_r^T + \mathcal{O}\left(\sum_{i=r+1}^{N_p} \frac{\lambda_i}{1 + \lambda_i} \right), \tag{S.5}$$

which corresponds to a low-rank correction of the prior covariance along the dominant data-informed directions. The remainder term quantifies the truncation error induced by neglected eigenmodes. A common criterion for controlling error is selecting r by discarding eigenvalues that satisfy $\lambda_{r+1} \ll 1$.

1.2 Laplace approximation of the model evidence

Bayesian model plausibility is quantified by the model evidence, which measures the probability of observing the data under a given model. In this work, we extended the Laplace

approximation to the posterior described in previous section, for scalable calculation of the posterior model plausibility. For notational simplicity, we omit explicit conditioning on the model index.

Under the Laplace approximation, rearranging Bayes' rule gives the following approximation of the log evidence,

$$\log \pi_{\mathrm{evid}}(d) \approx \log \pi_{\mathrm{like}}(d \mid \boldsymbol{\theta}_{\mathrm{MAP}}) + \log \pi_{\mathrm{pr}}(\boldsymbol{\theta}_{\mathrm{MAP}}) - \log \pi_{\mathrm{post}}(\boldsymbol{\theta}_{\mathrm{MAP}} \mid d). \tag{S.6}$$

For a Gaussian likelihood with data misfit

$$\Phi(\boldsymbol{\theta}) = \frac{1}{2}\left\|\phi(\mathbf{X};\boldsymbol{\theta}) - d(\mathbf{X})\right\|^2_{\boldsymbol{\Gamma}_{\mathrm{noise}}^{-1}}.$$

Then the log-likelihood evaluated at the MAP in (S.6) is obtained as,

$$\log \pi_{\mathrm{like}}(d \mid \boldsymbol{\theta}_{\mathrm{MAP}}) = -\Phi(\boldsymbol{\theta}_{\mathrm{MAP}}) - \frac{N_d}{2}\log(2\pi) - \frac{1}{2}\log|\boldsymbol{\Gamma}_{\mathrm{noise}}|. \tag{S.7}$$

Additionally, for a Gaussian random field prior,

$$\log \pi_{\mathrm{pr}}(\boldsymbol{\theta}_{\mathrm{MAP}}) = -\frac{1}{2}\left\|\boldsymbol{\theta}_{\mathrm{MAP}} - \bar{\boldsymbol{\theta}}\right\|^2_{\boldsymbol{\Gamma}_{\mathrm{pr}}^{-1}} - \frac{N_p}{2}\log(2\pi) - \frac{1}{2}\log|\boldsymbol{\Gamma}_{\mathrm{pr}}|, \tag{S.8}$$

and under the Laplace approximation, the posterior at the MAP is given by

$$\log \pi_{\mathrm{post}}(\boldsymbol{\theta}_{\mathrm{MAP}} \mid d) \approx -\frac{N_p}{2}\log(2\pi) - \frac{1}{2}\log|\boldsymbol{\Gamma}_{\mathrm{post}}|. \tag{S.9}$$

Here, N_p denotes the dimension of the discretized parameter fields over the finite element mesh, and N_d denotes the total number of scalar observation degrees of freedom in the discretized measurement field over the spatial domain. Substituting these expressions into (S.6) and canceling common terms yields

$$\begin{aligned}\log \pi_{\mathrm{evid}}(d) \approx\; & -\Phi(\boldsymbol{\theta}_{\mathrm{MAP}}) - \frac{1}{2}\left\|\boldsymbol{\theta}_{\mathrm{MAP}} - \bar{\boldsymbol{\theta}}\right\|^2_{\boldsymbol{\Gamma}_{\mathrm{pr}}^{-1}} \\ & - \frac{N_d}{2}\log(2\pi) - \frac{1}{2}\log|\boldsymbol{\Gamma}_{\mathrm{noise}}| + \frac{1}{2}\log|\boldsymbol{\Gamma}_{\mathrm{post}}| - \frac{1}{2}\log|\boldsymbol{\Gamma}_{\mathrm{pr}}|.\end{aligned} \tag{S.10}$$

Assuming independent, homoscedastic observation noise,

$$\boldsymbol{\Gamma}_{\mathrm{noise}} = \sigma^2_{\mathrm{noise}}\boldsymbol{I}, \qquad \log|\boldsymbol{\Gamma}_{\mathrm{noise}}| = N_d \log(\sigma^2_{\mathrm{noise}}).$$

Now, using the low-rank posterior covariance approximation in (S.5), the determinant ratio can be evaluated using Sylvester's identity and factoring out the prior covariance,

$$|\boldsymbol{\Gamma}_{\mathrm{post}}| \approx |\boldsymbol{\Gamma}_{\mathrm{pr}}|\left|\boldsymbol{I}_r - \boldsymbol{D}_r\boldsymbol{W}_r^T\boldsymbol{\Gamma}_{\mathrm{pr}}^{-1}\boldsymbol{W}_r\right|. \tag{S.11}$$

Using the $\boldsymbol{\Gamma}_{\mathrm{pr}}^{-1}$-orthonormality of the eigenvectors, $\boldsymbol{W}_r^T\boldsymbol{\Gamma}_{\mathrm{pr}}^{-1}\boldsymbol{W}_r = \boldsymbol{I}_r$, this expression simplifies to

$$\frac{|\boldsymbol{\Gamma}_{\mathrm{post}}|}{|\boldsymbol{\Gamma}_{\mathrm{pr}}|} = \left|\boldsymbol{I}_r - \boldsymbol{D}_r\right| = \prod_{i=1}^{r}\left(1 - \frac{\lambda_i}{1+\lambda_i}\right) = \prod_{i=1}^{r}\frac{1}{1+\lambda_i}, \tag{S.12}$$

and taking the logarithm yields

$$\log|\boldsymbol{\Gamma}_{\text{post}}| = \log|\boldsymbol{\Gamma}_{\text{pr}}| - \sum_{i=1}^{r} \log(1+\lambda_i). \tag{S.13}$$

Finally, combining all terms, the log-evidence can be approximated by

$$\log \pi_{\text{evid}}(d) \approx -\Phi(\boldsymbol{\theta}_{\text{MAP}}) - \frac{1}{2}\left\|\boldsymbol{\theta}_{\text{MAP}} - \bar{\boldsymbol{\theta}}\right\|^2_{\boldsymbol{\Gamma}_{\text{pr}}^{-1}} - \frac{N_d}{2}\log\left(2\pi\sigma^2_{\text{noise}}\right) - \frac{1}{2}\sum_{i=1}^{r}\log(1+\lambda_i). \tag{S.14}$$

This formulation enables scalable evaluation of posterior model plausibility in high-dimensional PDE-constrained Bayesian inference, with computational cost governed primarily by the number of retained data-informed modes and the associated forward and adjoint finite element solves, rather than scaling directly with the full parameter dimension.

2 Additional verification analyses

2.1 Sensitivity of model plausibility to fixed parameters

The model parameters C and H of biomechanical tumor growth model are fixed in the main analyses because they are weakly identifiable from MRI data. To assess whether the model plausibility results are sensitive to these prescribed values, we performed a sensitivity study for Rat III at the final MRI time (Day 19). Specifically, C and H were independently perturbed by ±25% relative to their baseline values used in the manuscript, while all other inference settings were kept unchanged. As shown in Table S1, the posterior plausibility values change only slightly under these perturbations, and the hyperelastic model remains more plausible than the linear elastic model. These results indicate that the model-plausibility trend is stable with respect to moderate perturbations of C and H.

Table S1: Sensitivity of posterior model plausibility to ±25% perturbations of the fixed model parameter C and H for Rat III at Day 19. The baseline values used in the main manuscript are $C = 0.65$ and $H = 1.5$.

Parameter	Value	ρ_{HE}	ρ_{LE}
C	0.4875	0.5061	0.4939
	0.65 (baseline)	0.5087	0.4913
	0.8125	0.5118	0.4882
H	1.125	0.5092	0.4908
	1.5 (baseline)	0.5087	0.4913
	1.875	0.5080	0.4919

2.2 Sensitivity of model plausibility to Hessian truncation rank

The Laplace-approximated model evidence depends on the retained dominant generalized Hessian eigenmodes. In the results presented in the main manuscript, we retained 50 modes based on the truncation error described in (S.5). To verify that the model plausibility results are not sensitive to this truncation choice, we repeated the evidence calculation for Rat III using 60 modes at two observation days. As shown in Table S2, increasing the retained rank from 50 to 60 did not change the posterior model plausibility values to the reported precision. This indicates that the evidence calculation is numerically stable with respect to the Hessian truncation rank.

Table S2: Sensitivity of posterior model plausibility to the number of retained Hessian eigenmodes in the Laplace evidence approximation for Rat III at Days 16 and 19.

Observation Day	Hessian Eigenmodes	ρ_{HE}	ρ_{LE}	ρ_{RD}
Day 16	50 modes	0.4011	0.4024	0.1964
	60 modes (+10)	0.4011	0.4024	0.1964
Day 19	50 modes	0.4132	0.3991	0.1877
	60 modes (+10)	0.4132	0.3991	0.1877

2.3 Multi-step tumor growth prediction

The main manuscript focuses on one-scan-ahead prediction to evaluate sequential Bayesian assimilation. Here, we additionally examine multi-step prediction by propagating the inferred model beyond the next MRI without intermediate data assimilation in Rat I in Figure S1. For each assimilation time t_k, the model is initialized using the corresponding MAP estimate and used to predict all subsequent MRI scans. As expected, prediction accuracy decreases as the forecast horizon increases.

2.4 Slice selection and adjacent-slice inference

To justify the two-dimensional slice used in the main manuscript, we evaluated the MRI-derived normalized tumor area (NTA) across neighboring axial slices for Rat III. As shown in Fig. S2, slice 10 contains the largest tumor burden over the longitudinal imaging window and exhibits consistent temporal growth, supporting its use as a representative slice for the 2D model construction and Bayesian inference.

As an additional robustness check, Bayesian inference and dynamic model selection were repeated for the adjacent slice 11 of Rat III. This slice contains a smaller tumor cross-section than the representative slice 10, and the first visible tumor region appears at Day 12 rather than Day 10. Despite this difference in tumor onset and size, the main trends are preserved (Figure S4). The mechanically coupled models remain more plausible than the uncoupled RD model, whose plausibility decreases as additional imaging data are assimilated. The competition is again primarily between the linear elastic and hyperelastic

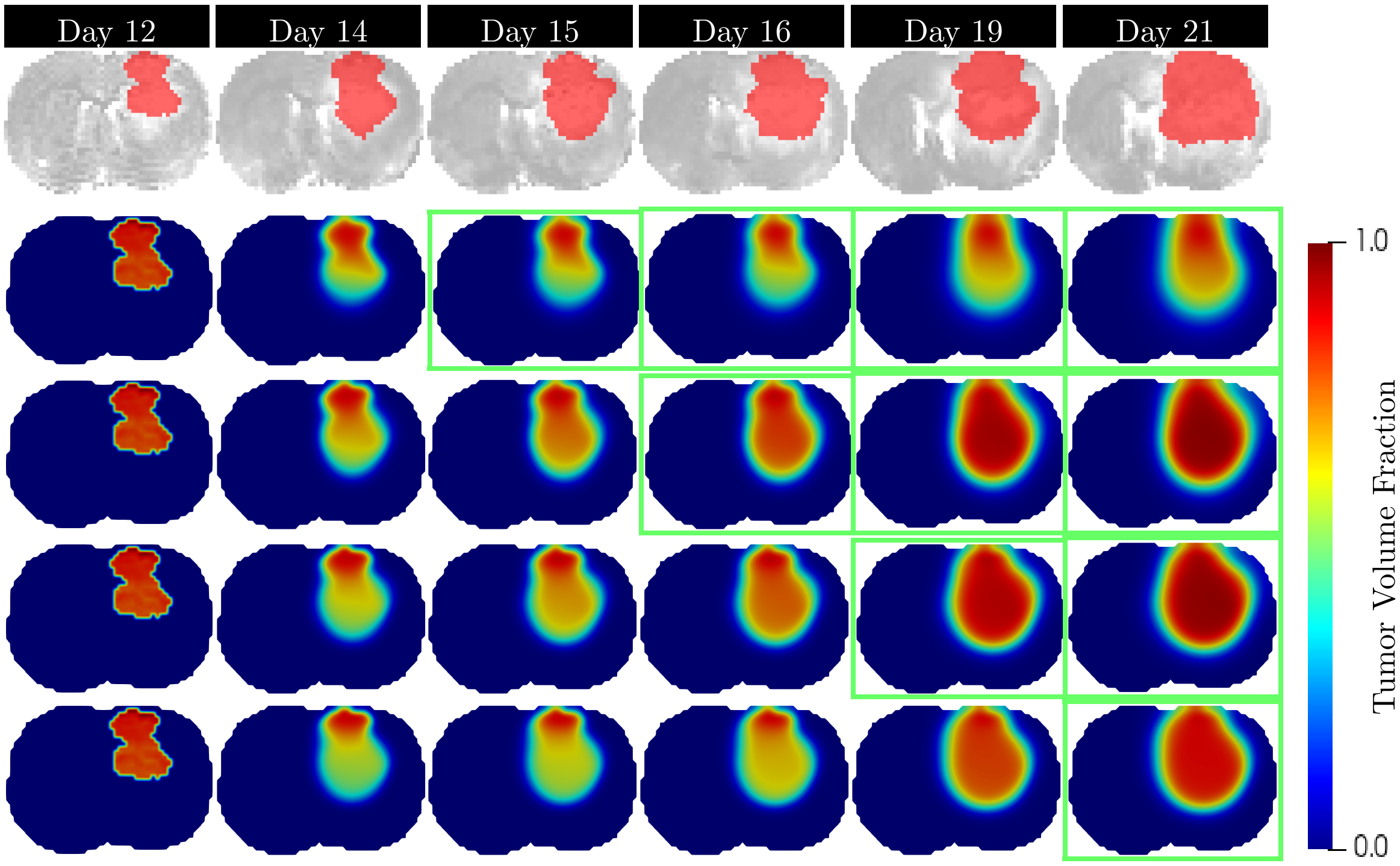

Figure S1: Multi-step tumor growth prediction for Rat I at MAP parameters. Top row: MRI-derived tumor volume fraction. Each lower row corresponds to predictions made after assimilating data up to a given time point and then propagating the model to all later scans without further updates. Boxed panels indicate future predictions. Dice values at the final prediction time, Day 21, are 0.85, 0.89, 0.91, and 0.92 for predictions initialized after assimilating data up to Days 14, 15, 16, and 19, respectively.

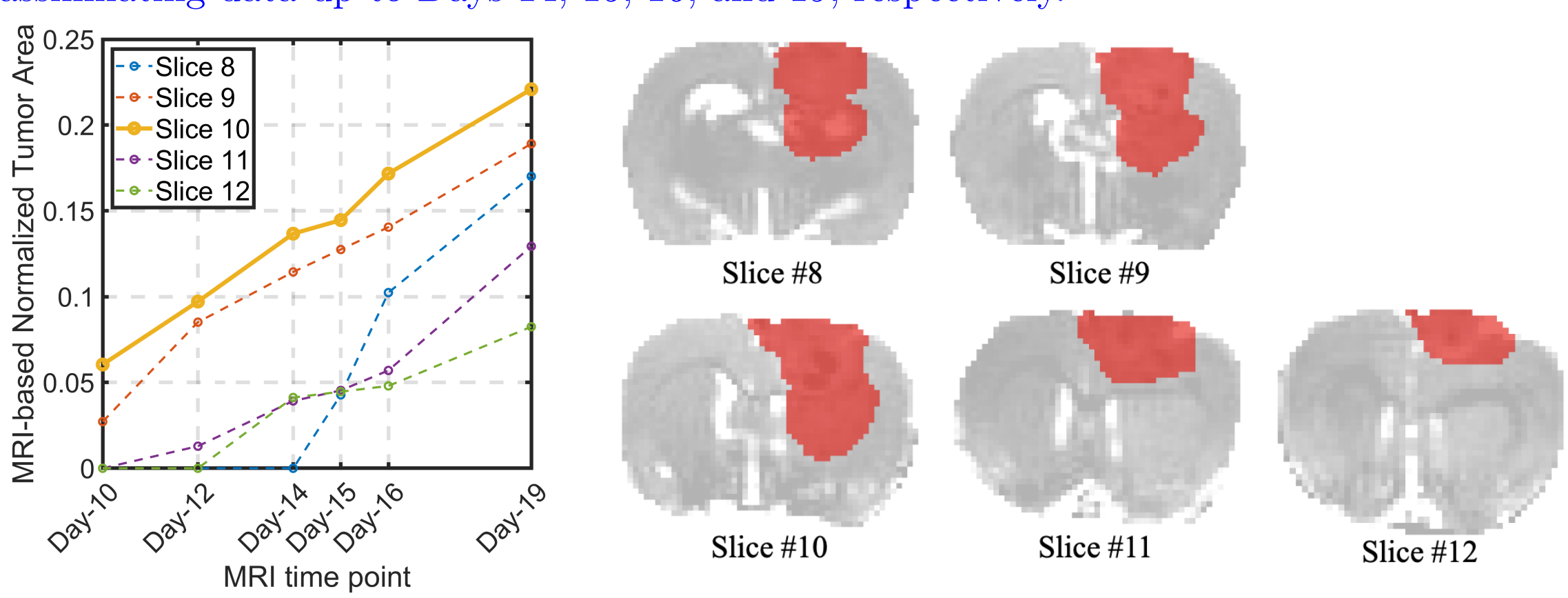

Figure S2: Slice-selection analysis for Rat-III. Left: MRI-derived normalized tumor area (NTA) over time for neighboring axial slices. Right: tumor boundaries for the corresponding slices at Day 19.

formulations, with the hyperelastic model becoming more favored at the later time point indicated by the NTA evolution plot. The Dice trends also remain broadly consistent with the slice 10 analysis, indicating that the main trend of higher plausibility for mechanically coupled models is not an artifact of selecting the central slice.

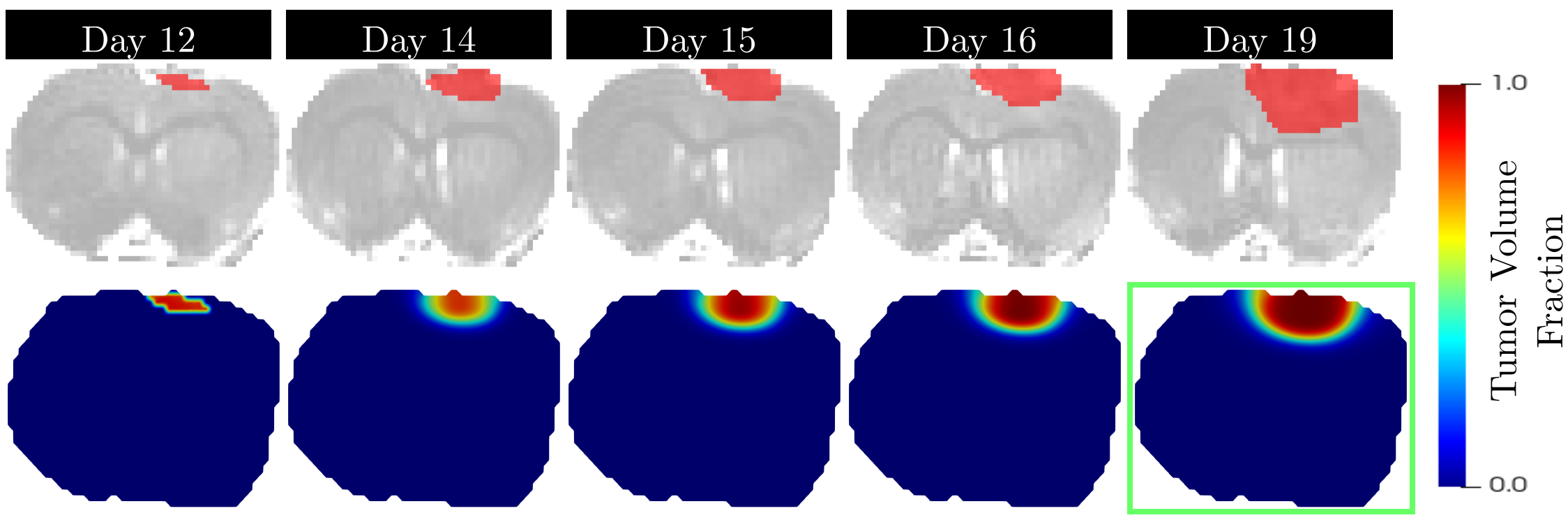

Figure S3: Tumor prediction for Rat III at MAP parameters with slice 11. Top row: MRI-derived tumor volume fraction. Bottom row: model predictions obtained by assimilating data up to each time point and predicting the subsequent scan (boxed panels).

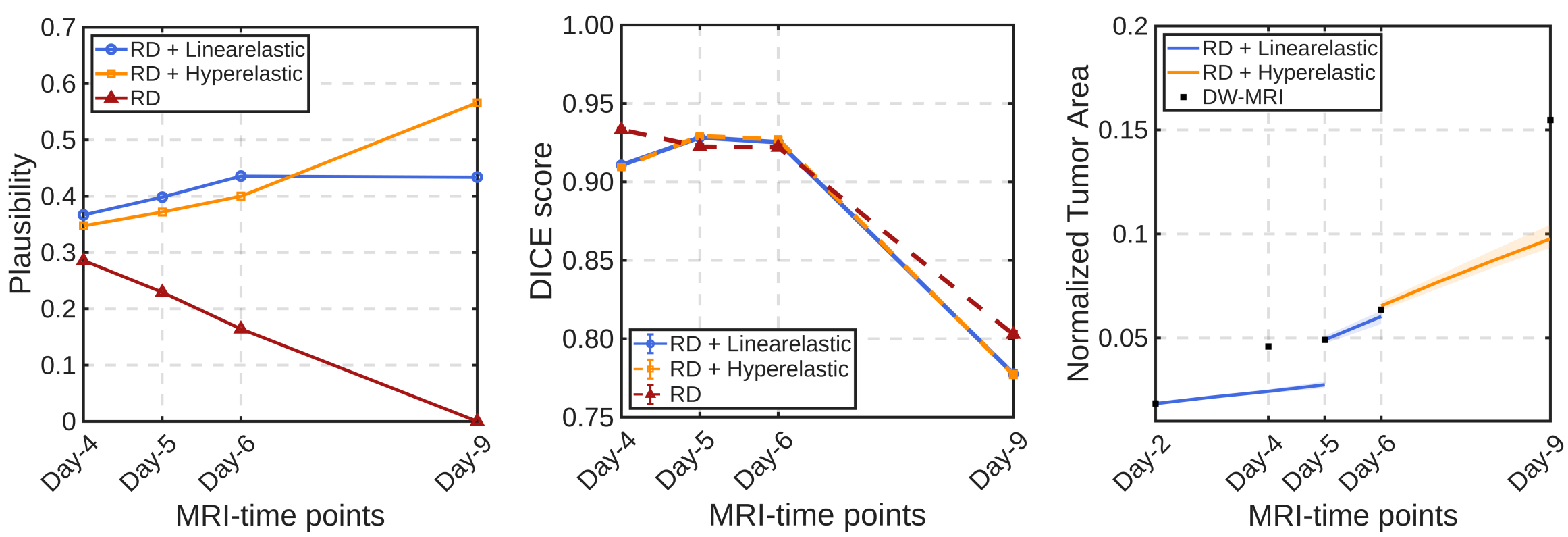

Figure S4: Temporal evolution of posterior model plausibility, one-scan-ahead predictive Dice score, and normalized tumor area (NTA) in Rat-III using Slice 11.

References

[1] T. Bui-Thanh and O. Ghattas. An analysis of infinite dimensional bayesian inverse shape acoustic scattering and its numerical approximation. *SIAM/ASA Journal on Uncertainty Quantification*, 2(1):203–222, 2014.

[2] T. Isaac, N. Petra, G. Stadler, and O. Ghattas. Scalable and efficient algorithms for the propagation of uncertainty from data through inference to prediction for large-scale problems, with application to flow of the antarctic ice sheet. *Journal of Computational Physics*, 296:348–368, 2015.

[3] U. Villa, N. Petra, and O. Ghattas. HIPPYlib: An Extensible Software Framework for Large-Scale Inverse Problems Governed by PDEs: Part I: Deterministic Inversion and Linearized Bayesian Inference. *ACM Trans. Math. Softw.*, 47(2), Apr. 2021.
